# *Saccharomyces* and non-*Saccharomyces* Competition during Microvinification under Different Sugar and Nitrogen Conditions

**DOI:** 10.3389/fmicb.2016.01959

**Published:** 2016-12-05

**Authors:** Jessica Lleixà, Maria Manzano, Albert Mas, María del C. Portillo

**Affiliations:** Biotecnología Enológica, Department Bioquímica i Biotecnologia, Facultat d‘Enologia, Universitat Rovira i VirgiliTarragona, Spain

**Keywords:** Torulaspora, Hanseniaspora, Starmarella, fermentation, wine

## Abstract

The inoculation of wines with autochthonous yeast allows obtaining complex wines with a peculiar microbial footprint characteristic from a wine region. Mixed inoculation of non-*Saccharomyces* yeasts and *S. cerevisiae* is of interest for the wine industry for technological and sensory reasons. However, the interactions between these yeasts are not well understood, especially those regarding the availability of nutrients. The aim of the present study was to analyze the effect of nitrogen and sugar concentration on the evolution of mixed yeast populations on controlled laboratory-scale fermentations monitored by density, plate culturing, PCR-DGGE and sugar and nitrogen consumption. Furthermore, the effect of the time of inoculation of *Saccharomyces cerevisiae* respect the initial co-inoculation of three non-*Saccharomyces* yeasts was evaluated over the evolution of fermentation. Our results have shown that *S. cerevisiae* inoculation during the first 48 h conferred a stabilizing effect over the fermentations with non-*Saccharomyces* strains tested and, generally, reduced yeast diversity at the end of the fermentation. On the other hand, nitrogen limitation increased the time of fermentation and also the proportion of non-*Saccharomyces* yeasts at mid and final fermentation. High sugar concentration resulted in different proportions of the inoculated yeast depending on the time of *S. cerevisiae* inoculation. This work emphasizes the importance of the concentration of nutrients on the evolution of mixed fermentations and points to the optimal conditions for a stable fermentation in which the inoculated yeasts survived until the end.

## Introduction

Wine is the result of alcoholic fermentation performed by yeasts during a complex process that transform the sugars present in the grape must into ethanol and carbon dioxide. During this alcoholic fermentation, a microbiological population evolves as a consequence of the chemical changes produced in the environment (Riberéau-Gayon et al., 2006). Many studies have established the yeast succession of non-*Saccharomyces* to *Saccharomyces* during spontaneous fermentation of grape juice. These non-*Saccharomyces* yeasts are the predominant microbiota in grapes and the main responsible for starting spontaneous alcoholic fermentation and often, under uncontrolled fermentations, lead to sluggish or stuck fermentations. For that reason, winemakers tend to inoculate grape must with commercial yeasts to ensure the completion of the fermentation, but compromising the complexity or the particular microbial footprint of wines of a certain region. In recent years, good properties and contribution of the non-*Saccharomyces* yeasts to wine and fermentation process have been described (Pretorius, 2000; Fleet, 2008; Ciani and Comitini, 2011; Jolly et al., 2014; Padilla et al., 2016a). With the aim to obtain wines that reflect a certain *terroir*, a previous study part of the WILDWINE project (Mas et al., 2016) accomplished the isolation and the characterization of multiple yeast strains from Priorat region to better understand the winemaking process and also to determine the source of microorganisms that produce a particular microbial footprint (Padilla et al., 2016b). The contribution of non-*Saccharomyces* takes part mostly during beginning and mid fermentation (Fleet, 2008). Non-*Saccharomyces* yeasts are able to produce metabolites or hydrolyze aromatic precursors providing new wine styles and enhancing their complexity (Ciani et al., 2010; Viana et al., 2011; Andorrà et al., 2012; Jolly et al., 2014).

The possibility to obtain wines with differential characteristics due to the role of non-*Saccharomyces* yeasts explains the increasing interest of using mixed cultures. As we have mentioned, one of the objectives of the WILDWINE project is to mimic the natural microbiota of a vineyard by the use of mixed inocula to perform fermentations to fight the wine uniformity derived from the widespread use of commercial *S. cerevisiae* starter cultures (Mas et al., 2016). Besides, interaction between non-*Saccharomyces* and *S. cerevisiae* has not been extensively studied, however some positive metabolic interactions have been described (Ciani et al., 2010; Ciani and Comitini, 2015). In the present study, the most characteristic non-*Saccharomyces* yeast isolated during the WILDWINE project were subjected to mixed alcoholic fermentation under different nutrient conditions (Mas et al., 2016; Padilla et al., 2016b).

The main problems during mixed fermentations are related to the nutrient composition of the must and the competition between the different yeast strains involved (Andorrà et al., 2010; Wang et al., 2015, 2016). It has been demonstrated that the consumption of nitrogen at the beginning of the fermentation by non-*Saccharomyces* yeast can prevent the correct development of *S. cerevisiae*.

Sugar and nitrogen composition of the grape must are key factors for the evolution of the alcoholic fermentation and the development of the yeasts (Bell and Henschcke, 2005; Beltran et al., 2005; Martínez-Moreno et al., 2012).

During the last few years, sugar content in grape must has become an important aspect since its concentration is increasing as a consequence of climate change and some viticultural practices (Mira de Orduña, 2010; Webb et al., 2012). The higher sugar content in grapes and, consequently, in musts is a problem for yeast physiology and it creates an osmotic stress that can produce, among others, stuck fermentations or wines with higher alcohol content.

In case of nitrogen, a higher or lower content can be harmful on fermentation kinetics and it has been demonstrated that a nitrogen concentration of 140 mg/L is the minimum required for yeasts to complete alcoholic fermentation (Bell and Henschcke, 2005), although this value is dependent on the sugar concentration (Martínez-Moreno et al., 2012). The same as sugar concentration, many factors can influence the nitrogen content on grapes and, consequently, on must such as environmental conditions and cultural practices (Bell and Henschcke, 2005).

The aim of this study was to determine the yeast dynamics and nutrient consumption during mixed fermentations of *Saccharomyces* and non-*Saccharomyces* yeast under four different nutrient conditions and with sequential addition of *S. cerevisiae* at four different time points. The fermentations were followed by density, plate culturing, PCR-DGGE and sugar consumption. According to our results, we propose the most suitable inoculation strategy for mixed fermentations using four strains isolated from Priorat region under the different nutrient concentrations.

## Materials and methods

### Yeast strains and starter cultures preparation

Four different yeast strains frequently isolated from natural must from Priorat Appellation of Origin (Catalonia, Spain) were employed (Padilla et al., 2016b). These yeasts were identified by ITS sequencing and identified and deposited in the Spanish Type Culture Collection (CECT) as *Saccharomyces cerevisiae* CECT 13132, *Hanseniaspora uvarum* CECT 13130, *Candida zemplinina* CECT 13129 (synonym: *Starmerella bacillaris*, Duarte et al., 2012) and *Toluraspora delbrueckii* CECT 13135. The starter cultures were prepared by growing the yeasts strains separately in liquid YPD medium (2% glucose, 2% Bacto peptone, 1% yeast extract, 2% agar, w/v; Cultimed, Barcelona, Spain) at 28°C with a stirring rate of 150 rpm in an orbital shaker.

### Mixed inoculum conditions

Fermentations were carried out in 250 mL of synthetic grape must (pH 3.3) as described by Riou et al. (1997), but with some modifications. The final concentration of sugars was either 200 or 240 g/L (denominated 200S or 240S, respectively) with a combination of glucose and fructose of 100 or 120 g/L each. The available nitrogen was either 100 or 300 mg/L (denominated 100N or 300N, respectively). Another variable was the time of the inoculation of *S. cerevisiae*: co-inoculation (0D), at 24 h (1D), at 48 h (2D) and at the 5th day (5D) after the inoculation of the non-*Saccharomyces*. Also, control fermentations were conducted for each nutrient condition with the sole inoculation of *S. cerevisiae*. Fermentations were considered finished when density was below 1000 g/L, or without variation for three consecutive days.

All the fermentations were performed in duplicate and inoculated at a concentration of 1.2·10^6^ cells/mL of *H. uvarum*, 5·10^5^ cells/mL of *S. bacillaris*, 1·10^5^ cells/mL of *T. delbrueckii* and 2·10^6^ cells/mL of *S. cerevisiae*. These concentrations resemble yeast populations of natural musts from Priorat, where the non-*Saccharomyces* yeasts were isolated (Wang et al., 2015) and the practice of inoculating commercial *Saccharomyces* presentations.

### Density, acetic acid, and sugar measurements

The fermentations were monitored daily by density with Densito 30PX Portable Density Meter (Mettler Toledo, Spain). Once the fermentations were finished (the density was under 1000 g/L or stable for 3 days), concentrations of glucose and fructose and the acetic acid concentration in the final fermentation samples were analyzed by Miura One Multianalyzer (TDI, Barcelona, Spain) using the enzymatic kit from Biosystems S. A. (Barcelona, Spain). Samples for plating, qPCR and PCR-DGGE were taken at the beginning (24 h after incubation started), in the middle (density approximately 1020–1030 g/L) and at the end of fermentation (density below 1000 g/L or stable for 3 days). Maximum fermentation rate (R) was calculated as maximum slope of the density measurements respect the time. Also, time to reach the 10, 50, and 75% of the final density (referred as t10, t50, and t75, respectively) were calculated as additional parameters of the fermentation kinetics (Table S1). Successful fermentations were considered when density was below 1000 and residual sugar was below 3 g/L.

### Plate culturing

Fresh samples were directly analyzed by culture-dependent techniques at each fermentation stage (beginning, middle and end of fermentation). The total yeast populations were enumerated on plates with YPD medium. The Wallerstein Laboratory nutrient agar (WL; Oxoid, England) is useful to quantify and identify wine microorganisms and was used to discriminate between the used yeast species by colony morphology and color (Pallmann et al., 2001).

### DNA extraction

Cell pellets from 1 mL of samples at each fermentation stage (beginning, middle and end of fermentation) were collected by centrifugation after washing with sterile water and kept at −80°C for further culture-independent analysis by and PCR-DGGE. DNA cell pellets were extracted according to Hierro et al. (2007). The concentration and purity of DNA was determined using a NanoDrop 1000 spectrophotometer (Thermo Fisher Scientific, Wilmington, DE, U.S.A.).

### PCR-DGGE analysis

The PCR reactions were performed using a Gene Amp PCR System 2720 (Applied Biosystems, USA) with Primers U1^GC^ and U2 (Meroth et al., 2003). The DGGE procedures followed the description in Andorrà et al. (2008) with a modified DGGE gel using a denaturing gradient from 35 to 55% urea and formamide. A marker prepared with the PCR products of each individual yeast species was included in the DGGE gels for migration comparison and yeasts identifications.

### Statistical analysis

Fermentation kinetics variables (residual sugar, acetic acid concentration, R, t10, t50, and t75) have been used to construct a dissimilarity matrix based on Euclidean distance between their values. All these variables have been used to construct a dissimilarity matrix based on the Euclidean distance between their values. ANOSIM (an analog of univariate ANOVA which tests for differences between groups of samples) was run in PRIMER v6 (Clarke and Gorley, 2006) to determine significant differences between the different fermentations among the main experimental factors (sugar and nitrogen content, residual sugar, *S. cerevisiae* inoculation time). Principal coordinate analysis (PCoA) was used to summarize and visualize the different fermentations under each Nitrogen condition respect the final residual sugar (as an estimator of fermentation success). Pearson correlation analysis were performed between the residual sugar and the rest of parameters.

## Results and discussion

### Effect of nutrients concentration on fermentation kinetics

Fermentations with optimal nitrogen concentration (300N-240S and 300N-200S) were all completed in 5–13 days, with the fermentations under excess of sugar (300N-240S) the slower ones (Table 1) (Figure S1). On the other hand, most of the fermentations performed under limiting nitrogen concentration (100N-240S and 100N-200S) got stuck (Table 1). From these results we observed that the nitrogen content had a stronger effect than the sugar concentration in yeast metabolism and affected the fermentation kinetics. Also, ANOSIM results showed that the fermentations under different nitrogen concentration (100N and 300N) were significantly different (Table 2), i.e., their kinetics parameters (R, t10, t50, t75, residual sugar and acetic acid) were different for each nitrogen condition. However, sugar concentration (200S and 240S) did not result in significant differences (Table 2).

**Table 1:** **Evolution of the different fermentations (0D, co-inoculated fermentation; 1D, inoculation of *S. cerevisiae* at 24 h; 2D, inoculation of *S. cerevisiae* at 48 h; 5D, inoculation of *S. cerevisiae* at 5 days; and Control, only *S. cerevisiae*) under four nutrient conditions (300N-200S, 300N-240S, 100N-200S, and 100N-240S)**.

**Nutrient condition**	**Inoculation time**	**MF (days)**	**EF (days)**	**BF (CFU/mL)**	**MF (CFU/mL)**	**EF (CFU/mL)**	**Residual sugar (g/L)**
300N	0D	3	5	4.0 ± 0.04E+06	6.7 ± 0.08E+07	3.9 ± 0.05E+07	4.87±0.21
	1D	5	7	3.2 ± 0.08E+06	2.7 ± 0.09E+07	2.0 ± 0.04E+07	0.01±0.01
200S	2D	5	8	3.2 ± 0.05E+06	4.1 ± 0.01E+07	4.8 ± 0.03E+07	Nd
	5D	4	6	7.1 ± 0.02E+06	3.9 ± 0.03E+06	3.0 ± 0.03E+06	10.18±0.37
	Control	3	5	5.6 ± 0.09E+06	7.5 ± 0.07E+07	2.5 ± 0.07E+07	0.01±0.01
300N	0D	5	7	8.4 ± 0.02E+06	2.9 ± 0.04E+08	1.9 ± 0.06E+08	5.52±0.37
	1D	5	9	5.3 ± 0.05E+06	5.0 ± 0.01E+07	3.2 ± 0.04E+07	2.80±0.14
240S	2D	7	13	3.0 ± 0.03E+06	4.0 ± 0.06E+07	2.3 ± 0.05E+07	Nd
	5D	7	12	8.5 ± 0.08E+06	2.3 ± 0.06E+08	1.2 ± 0.08E+08	30.90±0.71
	Control	3	5	2.0 ± 0.05E+06	1.0 ± 0.09E+07	2.5 ± 0.03E+08	0.19±0.01
100N	0D	5	8	8.0 ± 0.05E+06	7.4 ± 0.07E+07	5.4 ± 0.05E+07	0.32±0.01
	1D	6	−	4.8 ± 0.07E+06	1.8 ± 0.04E+07	1.4 ± 0.05E+07	43.80±3.68
200S	2D	6	−	4.2 ± 0.08E+06	2.7 ± 0.05E+07	7.6 ± 0.04E+06	53.80±4.38
	5D	6	−	3.1 ± 0.03E+06	7.2 ± 0.06E+06	3.8 ± 0.04E+06	57.50±2.62
	Control	5	8	3.0 ± 0.08E+06	1.1 ± 0.06E+07	7.4 ± 0.03E+06	Nd
100N	0D	7	−	3.9 ± 0.05E+06	2.4 ± 0.04E+07	2.6 ± 0.02E+07	13.20±0.57
	1D	7	−	3.4 ± 0.04E+06	3.0 ± 0.05E+07	9.9 ± 0.04E+06	51.10±0.49
240S	2D	11	−	2.1 ± 0.04E+06	9.6 ± 0.02E+06	9.8 ± 0.02E+06	40.40±3.25
	5D	11	−	2.4 ± 0.08E+06	1.7 ± 0.06E+07	2.0 ± 0.04E+07	64.40±2.76
	Control	5	7	3.3 ± 0.03E+06	1.1 ± 0.04E+07	8.8 ± 0.05E+06	19.30±0.92

*Results expressed as days spent to reach the middle (MF) and the end of the fermentation (EF), population growth in YPD at the beginning (BF), middle (MF) and end of the fermentation (EF) and the residual sugar (glucose+fructose) measured at the end of the fermentation or, when density was stable for three consecutive days, the last point was considered*.

**Table 2:** **ANOSIM of the different factors effect on the fermentations based on a dissimilarity matrix calculated by the Euclidian distance of the kinetic parameters**.

**Samples**	**Factor**	**R**	***P***
All	Nitrogen	**0.402**	**0.001**
All	Sugar	0.036	0.15
All	Inoculation time	**0.243**	**0.001**
All	Residual sugar	**0.864**	**0.001**
All	Succ. fermentation	**0.561**	**0.001**

*Values of statistical significance (P) below 0.05 (bold values) indicate significantly different fermentations considering a certain factor. Successful fermentation was considered when the residual sugar was below 3 g/L*.

**Figure 1:**
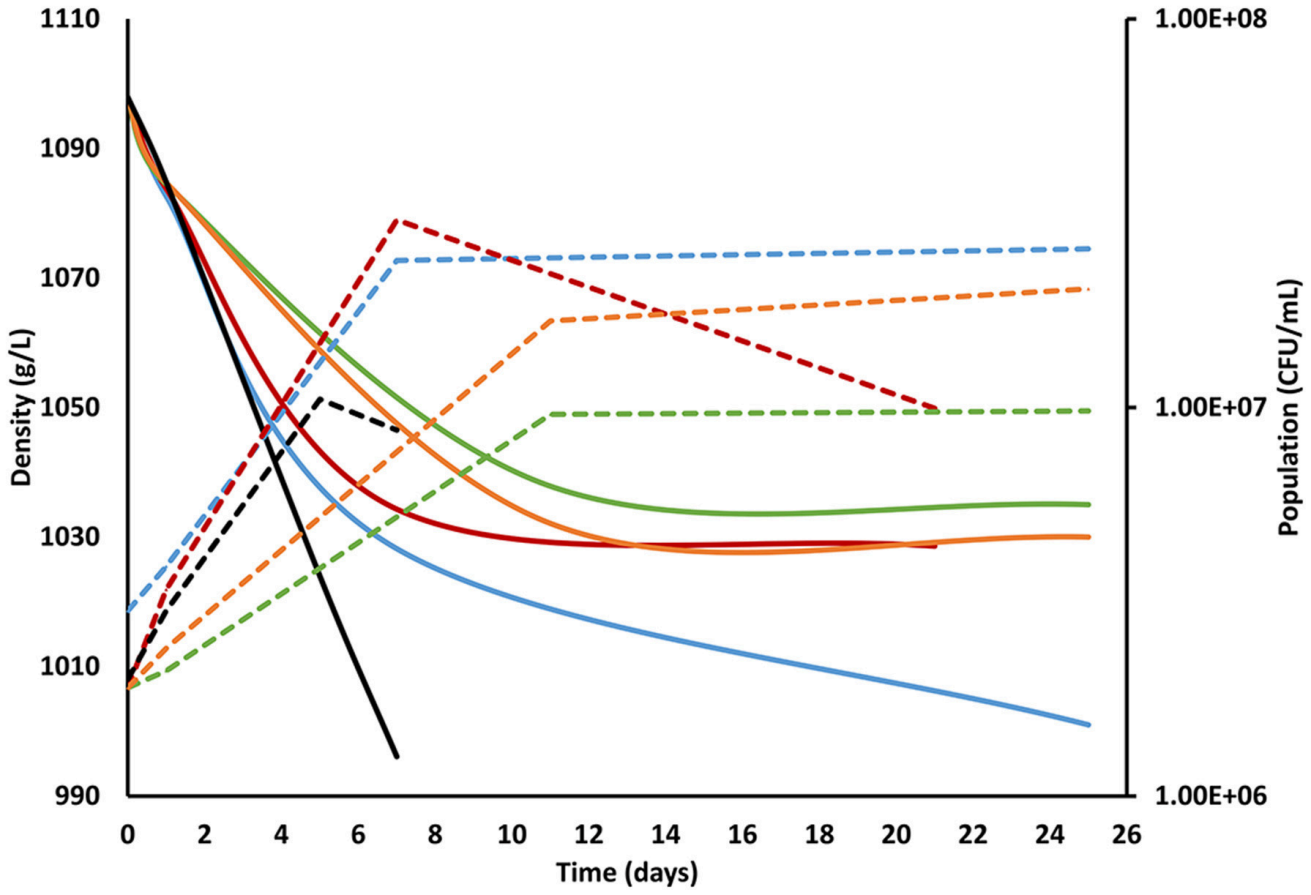
**Fermentation kinetics of the different inoculation strategies performed under 100N-240S nutrient conditions**. The solid line shows the evolution of the fermentation measured by density (g/L) and the dotted line assessed by plate culturing in YPD (CFU/mL). The line color corresponds to each fermentation strategy: blue, co-inoculated fermentation; red, inoculation of *S. cerevisiae* at 24 h; green, inoculation of *S. cerevisiae* at 48 h; orange, inoculation of *S. cerevisiae* at 5 days, and black; control fermentation with only *S. cerevisiae*. Standard deviations were always lower than 10% and have been avoided in the figure for clarity.

It has been previously described that nitrogen concentration below 140 mg/L are limiting to growth and result in a decrease of the fermentation rate by *S. cerevisiae*, an increase the risk of sluggish and stuck fermentation as well as an increase in residual sugars (Bell and Henschcke, 2005; Martínez-Moreno et al., 2012; Tesnière et al., 2015). However, according to our results, both 100N control fermentations inoculated just with *S. cerevisiae* were able to be completed in 7–8 days (Table 1). This could be explained by the different nitrogen requirements of the selected *S. cerevisiae* strain, autochthonous yeast that was grown in YPD before its inoculation in the synthetic must, thus allowing inner nitrogen accumulation. Mixed fermentations with the four yeast species, with expected different nitrogen and sugar requirements, got generally stuck under 100N and it would be interesting to investigate the required addition of nitrogen to complete those fermentations (Table 1) (Figure 1). This could be due to the known higher nitrogen requirements of non-*Saccharomyces* yeast (Andorrà et al., 2010, 2012). The consumption of the available nitrogen by the non-*Saccharomyces* yeasts and the delay in *S. cerevisiae* inoculation could increase the risk of stuck and sluggish fermentations (Medina et al., 2012).

High-sugar must (240S) was indeed expected to result in longer fermentations since it has been previously described that high sugar concentration slows down yeasts growth and the progress of fermentation (Riberéau-Gayon et al., 2006). It has been suggested that the main stress factor under high sugar conditions would be the ethanol content and not the sugar osmotic pressure (Nishino et al., 1985; Mauricio and Salmon, 1992). Bisson and Butzke (2000) observed that a nitrogen supplementation could be appropriate in fermentations with *S. cerevisiae* under 240 g/L of sugar to complete the fermentation and Martínez-Moreno et al. (2012) suggested that 160 mg/L of nitrogen would be the minimum requirement at this sugar concentration. Conversely, other authors demonstrated in *S. cerevisiae* that the addition of nitrogen in high-sugar musts did not necessarily lead to complete fermentations even taking into account the nitrogen utilization requirements by different strains of *S. cerevisiae* (Martínez-Moreno et al., 2012; Childs et al., 2015). According to our results, a supplementation of 300 mg/L of nitrogen was enough to finish all the 240S fermentations.

### Effect of sequential inoculation of *S. cerevisiae* over fermentation kinetics

**Figure 2:**
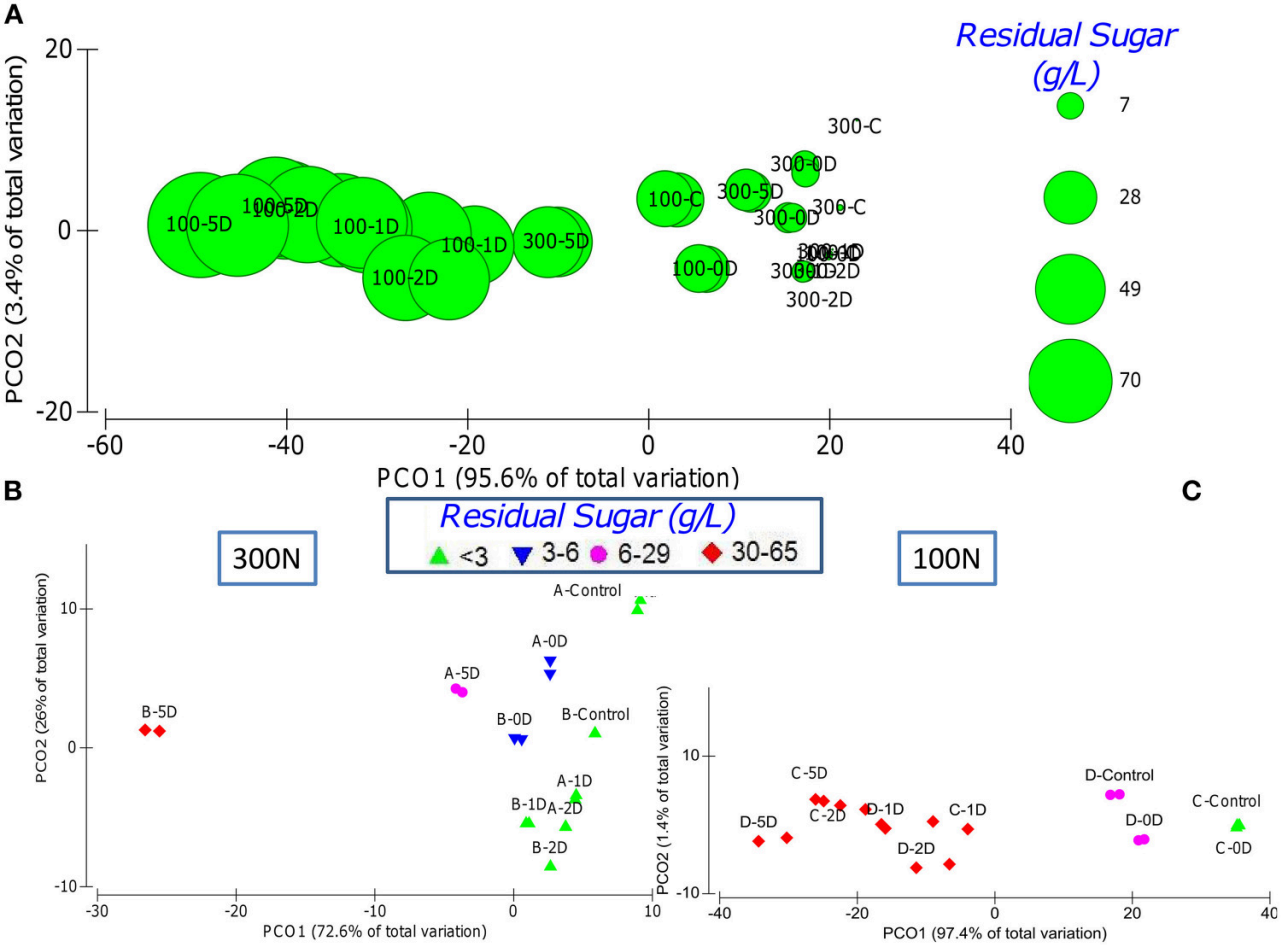
**PCA graphs displaying the dissimilarity between the different fermentations taking into account the kinetics parameters R, t10, t50, t75, acetic acid and residual sugar. (A)** PCA representing all the fermentations respects the residual sugar content (proportional to the bubbles size) with the clustering of most of 100N fermentations at the left and most of the 300N fermentations at the right. PCA of the 300N **(B)** and 100N **(C)** fermentations respect the residual sugar where the initial sugar concentration is indicated by A or C (200S) and B or D (240S), Control represent the inoculations with only *S. cerevisiae* and the inoculation time of *S. cerevisiae* is indicated by 0D, 1D, 2D, and 5D.

The inoculation time of *S. cerevisiae* have a significant impact over the fermentation kinetics parameters (Table 1), especially within each nitrogen concentration (Figures 2A,B). Control fermentations performed just with *S. cerevisiae* were the fastest to complete (5–8 days) under any of the nutrient conditions and only matched by co-inoculation (0D) under optimal sugar concentrations (300N-200S and 100N-200S).

**Figure 3:**
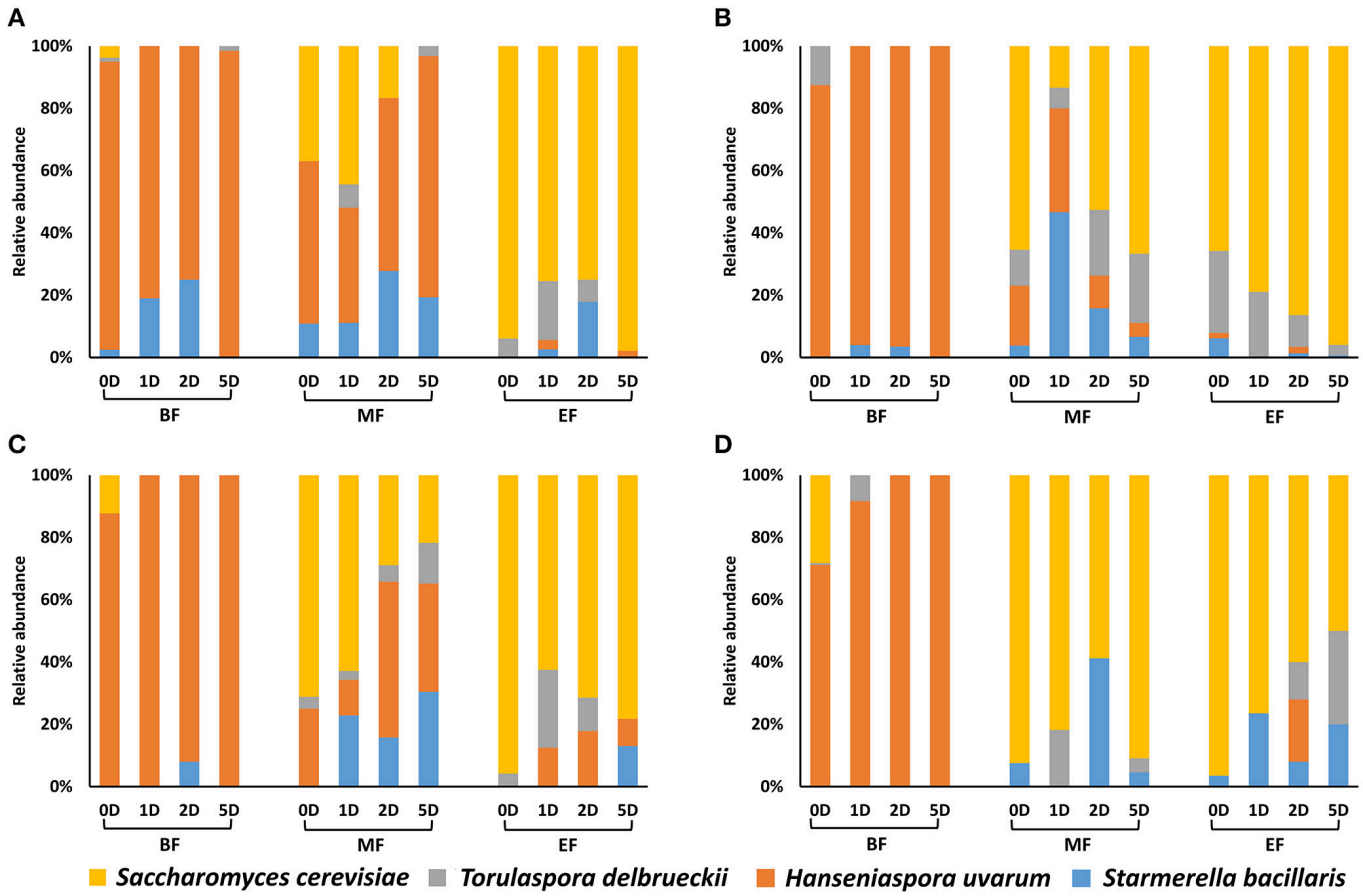
**Yeast population dynamics at the beginning (BF), middle (MF) and end of the fermentation (EF) under four different nutrient conditions, (A)** 300N-200S, **(B)** 300N-240S, **(C)** 100N-200S, and **(D)** 100N-240S. The fermentations strategies were: 0D, co-inoculated fermentation; 1D, inoculation of *S. cerevisiae* at 24 h; 2D, inoculation of *S. cerevisiae* at 48 h; and 5D, inoculation of *S. cerevisiae* at 5 days.

Under optimal nitrogen concentration (300N), the sequential inoculation of *S. cerevisiae* from 24 h onward had different effect over the fermentation kinetics depending on the sugar concentration. However, the earlier inoculation of *S. cerevisiae* did not imply that fermentation finished faster (Table 1). For example, it is interesting to observe that fermentations where *S. cerevisiae* was inoculated at 24–48 h (1D, 2D) under a nitrogen concentration of 300 mg/L took longer to finish than those where *S. cerevisiae* was added 5 days after the beginning of the fermentation (Table 1; Figure S1). This result was also reflected in the separation of these samples from the rest of the 300N samples as a consequence of the differences in the fermentation kinetics parameters (Table 2, Figure 2B). A possible explanation could be that at day 5, when *S. cerevisiae* was inoculated, half of the fermentation had already been spent and the viable non-*Saccharomyces* yeast were decreasing (Table 1, Figure 3) which meant less competition for nutrients by *S. cerevisiae*. Additionally, the death and the autolysis of non-*Saccharomyces* yeast could result in an extra nitrogen source for *S. cerevisiae* (Hernawan and Fleet, 1995).

Under limiting nitrogen concentration (100N), as stated in the previous section, most of the fermentations got stuck and have a high residual sugar (Table 1, Figures 2A,C). However, control fermentations were able to finish and, under optimal sugar conditions, the co-inoculation of *S. cerevisiae* and the three non-*Saccharomyces* allowed the fermentation to complete as well (Table 1). These results allowed the separation of these fermentations from the rest fermentations on the PCA analysis taking into account all the kinetics parameters (Figure 2C). Some authors have proved that co-inoculated fermentations with one or two non-*Saccharomyces* yeast species are a good strategy to ensure *S. cerevisiae* development and the fermentation process (Andorrà et al., 2010; Medina et al., 2012). According to our results, the time of *S. cerevisiae* inoculation acquired more importance under limiting nitrogen content as a consequence of nutrient consumption by the different yeasts species. Medina et al. (2012) demonstrated that an increase of the inoculum size of non-*Saccharomyces* yeasts or the inoculation of *S. cerevisiae* after 24 h decreases the growth of the latter and slowed the fermentation rate of the mixed fermentation as a consequence of the nutrient consumption by non-*Saccharomyces* yeasts.

Thus, a limiting nitrogen concentration together with a sequential inoculation of *S. cerevisiae* later than 48 h involves nitrogen consumption by non-*Saccharomyces* yeasts that limits *S. cerevisiae* development and the fermentation progress.

### Yeast dynamics by plate culturing and PCR-DGGE

Both culture dependent and independent techniques (plate culturing and PCR-DGGE) were used to follow yeast dynamics at each fermentation stage (beginning, the middle and the end of the fermentation). The differential morphology of the colonies on WL medium of the four selected yeast species allowed us to calculate the proportion of each cultivable yeast species at each fermentation stage (Figure 3). Moreover, to compare with molecular analysis results thus avoiding underestimation by the presence of viable but non cultivable (VBNC) yeast, we performed PCR-DGGE analysis of the extracted DNA at each fermentation stage using general yeast primers (Meroth et al., 2003).

Figure 3 and Table 3 show that the results obtained by these two techniques were usually comparable. However, as previous studies have reported (Andorrà et al., 2008, 2010) plate culturing proved to be more sensitive than using PCR-DGGE when the proportion of a specific species was very low at some fermentation stages. For example, by DGGE we could not detect *S. bacillaris* and *T. delbrueckii* in most of the fermentation stages while a little proportion of these species was recovered by plate-culturing technique in almost all fermentation stages and conditions. However, under nutrient limiting and sugar excess conditions (100N-240S) the DGGE technique was more efficient and we were able to detect higher yeast diversity maybe as a consequence of the loss of yeast cultivability under these extreme conditions (Table 3).

**Table 3:** **Results of the DGGE-PCR for *H. uvarum* (Hu), *S. bacillaris* (Sb), *T. delbrueckii* (Td) and *S. cerevisiae* (Sc) expressed as “++” (the intensity of the band detected by DGGE gel was high), “+” (the intensity of the band detected by DGGE gel was weak) and “−” (no band was detected by DGGE gel)**.

**Nutrient condition**	**Inoculation time**	**Beginning fermentation**				**Middle fermentation**				**End fermentation**			
		**Hu**	**Sb**	**Td**	**Sc**	**Hu**	**Sb**	**Td**	**Sc**	**Hu**	**Sb**	**Td**	**Sc**
300N	0D	++	−	−	−	+	−	−	−	−	+	+	++
	1D	++	−	−	−	++	+	+	++	−	+	+	++
200S	2D	++	−	−	−	++	−	−	+	−	−	−	+
	5D	++	−	−	−	+	−	−	−	−	−	−	+
300N	0D	+	−	−	−	−	+	+	++	−	+	+	++
	1D	++	−	+	−	++	−	+	+	−	+	−	++
240S	2D	++	−	+	−	−	+	−	++	−	−	−	++
	5D	++	−	−	−	++	−	+	++	−	−	+	+
100N	0D	+	−	−	+	+	−	−	++	−	−	−	++
	1D	+	+	−	−	++	−	+	+	−	−	−	+
200S	2D	++	+	−	−	++	−	+	+	+	−	−	++
	5D	+	−	+	−	++	−	−	−	−	−	−	+
100N	0D	++	−	−	+	+	+	+	++	−	−	−	++
	1D	++	−	−	−	++	−	−	+	+	−	−	++
240S	2D	++	−	−	−	−	+	+	++	−	+	+	++
	5D	++	−	−	−	+	+	+	++	−	−	−	++

The main yeast species at the beginning of the fermentation (24 h) in all cases was *H. uvarum* while, at the end of the fermentation *S. cerevisiae* took over. We used a higher inoculum of *H. uvarum* compared to the other non-*Saccharomyces*, as occurs on natural must from the Priorat DOQ region (Wang et al., 2016), and this would explain the *H. uvarum* high proportion at the beginning of the fermentation respect to *S. bacillaris* and *T. delbrueckii*. In this sense, our results are similar to those obtained in spontaneous grape fermentations where *H. uvarum* was in great proportion at the first stages of the fermentation in Priorat area (Constantí et al., 1998; Torija et al., 2001; Wang et al., 2016).

It is interesting that a low proportion of *S. cerevisiae* was recovered at the beginning of all the fermentations even when it was co-inoculated with the non-*Saccharomyces* even taking into account that its inoculum size was similar to that of *H. uvarum*. Previous studies have reported that the initial growth of *H. uvarum* retarded the growth of *S. cerevisiae* (Herraiz et al., 1990) which could be an explanation of this effect.

In the middle of the fermentation the yeast species proportion deeply varies depending on the nutrients and the time of inoculation of *S. cerevisiae* (Figure 3). For example, under optimal nutrient conditions (300N-200S) at the mid fermentation, the non-*Saccharomyces* yeasts overgrew *S. cerevisiae* that was just more abundant at inoculation 0D or 1D (37 and 44.4%, respectively). Medina et al. (2012) noticed a negative effect of non-*Saccharomyces* yeast on nutrient availability for *S. cerevisiae* reducing its ability for grow especially when it was sequentially inoculated. Interestingly, when they added nitrogen supplementation the fermentation rate and the proportion of *S. cerevisiae* increased, this effect was more prominent when they added a supplement of YAN and vitamin. This YAN consumption by non-*Saccharomyces* yeasts would explain the low imposition of *S. cerevisiae* over the different fermentations at the middle of the fermentation, specifically when *S. cerevisiae* was inoculated 24 h and after. However, under excess of sugar (300N-240S), *S. cerevisiae* was the most frequently recovered at 0D, 2D and 5D (52.6–66.6%) being in low proportion at 1D when the non-*Saccharomyces* yeasts (mainly *S. bacilaris*) represented more than 80%. Thus, at 300N-240S *S. cerevisiae* was able to overtake non-*Saccharomyces* yeasts at the middle of the fermentation except when it was inoculated at 24 h although the non-*Saccharomyces* yeasts were present in the mid fermentation under any of the conditions contemplated in the present study. We also observed that the excess of sugar (240S) affected negatively to *H. uvarum* respect the 200S conditions. Under nitrogen limitation (100N-200S/240S), we recovered higher proportion of *S. cerevisiae* at the middle of the fermentation than under the respective 300N fermentations.

At the end of the fermentation, *S. cerevisiae* was the most abundant yeast under any of the analyzed conditions, though *S. bacillaris* and *T. delbrueckii* were also present and generally in higher proportion than *H. uvarum*. In a previous study, Ciani et al. (2006) proved the high persistence of *H. uvarum* in mixed fermentations with *S. cerevisiae* under excess of sugar (270 g/L) and low temperature (15°C), which is in accordance with our results. Wang et al. (2016) demonstrated that *T. delbrueckii* and *S. bacillaris* where able to maintain its cultivability longer than *H. uvarum* when they were inoculated with *S. cerevisiae*. Furthermore, many interactions between non-*Saccharomyces yeasts* and *S. cerevisiae* can occur in the mixed fermentations under the studied conditions: yeast-yeast cell contact, antimicrobial compounds release or competition for substrate (Ciani and Comitini, 2015). It has been described that *S. cerevisiae* produce metabolites that negatively affect non-*Saccharomyces* yeasts (Pretorius, 2000; Pérez-Nevado et al., 2006; Wang et al., 2016). So, the effect of these metabolites together with the chemical changes on the medium could provide an explanation for the decrease of *H. uvarum* and the persistence and increase of *T. delbrueckii* and *S. bacillaris* along the fermentation, because the sensibility to these antimicrobial compounds is species and strain specific (Wang et al., 2016).

### Fermentation products

Total residual sugars were evaluated at the end of the fermentation or, in the case of stuck fermentations, at the last considered point with stable density for three consecutive days, using an enzymatic kit as described in Section Density, Acetic Acid, and Sugar Measurements. Residual sugars were significantly correlated with all the kinetic parameters considered except with the initial sugar concentration (Table S2).

Successful fermentations with residual sugar below 3 g/L where just those performed under optimal nitrogen concentration inoculated with *S. cerevisiae* at 48 H or before and under limiting nitrogen concentration when *S. cerevisiae* was the only yeast inoculated or when the non-*Saccharomyces* yeasts where co-inoculated (Figures 2B,C). These successful fermentations had kinetics parameters statistically different from the rest of fermentations tested (Table 2).

Fermentations performed under suitable nitrogen content (300N-200S/240S) presented the lowest residual sugars when they were sequentially inoculated at 24 or 48 h (Table 1). Unexpectedly, co-inoculated fermentations had a final sugar content between 4 and 6 g/l which could be explained by the high persistence of non-*Saccharomyces* yeast (Figure 3) that have been described as low fermentative yeasts (Pretorius, 2000). Besides, when *S. cerevisiae* was added after 5 days, sugar content was quite high as a consequence of the *S. cerevisiae* nutrient deprivation by non-*Saccharomyces* yeasts, which compromised its development and metabolic capacities (Andorrà et al., 2010; Medina et al., 2012).

On the other hand, under nitrogen limiting conditions (100N-200S/240S) the residual sugar concentration was very high at all fermentation stages as a consequence of the stuck fermentations resulting from the nutrient limitation (Bell and Henschcke, 2005) and just the co-inoculated fermentations (100N 200S) that completed the fermentation showed a lower residual sugar (Table 1).

## Conclusions

Nowadays, the use of mixed fermentations represents a powerful tool as a consequence of the combination of the positive abilities of non-*Saccharomyces* yeasts with *S. cerevisiae*. Despite this fact, nutrient must conditions and the time of the inoculation of *S. cerevisiae* can determine an adequate fermentation performance. We have demonstrated the negative impact of limiting nitrogen musts on mixed fermentation resulting in stuck fermentations with higher significance than sugar concentration. However, an excess of sugar must slowed down the fermentation rate. Furthermore, the best inoculation time of *S. cerevisiae*, under adequate nitrogen concentration would be before 48 h to ensure the completion of the fermentation due to the nitrogen consumption by non-*Saccharomyces*. However, inoculations before 24 h low the proportion of non-*Saccharomyces* yeasts that could contributed to the complexity of the wines. On the other hand, under nitrogen-limiting conditions, *S. cerevisiae* should be co-inoculated to ensure the fermentation process and the nitrogen availability for this yeast.

## Author contributions

JL performed part of the experiments, analyzed the results and draft the manuscript. MM performed part of the experiments. AM and MP conceived the study and participated in its design and coordination and draft the manuscript. All authors read and approved the final manuscript.

### Conflict of interest statement

The authors declare that the research was conducted in the absence of any commercial or financial relationships that could be construed as a potential conflict of interest.
