# Supplementary material for: Saccharomyces and non-Saccharomyces Competition during Microvinification under Different Sugar and Nitrogen Conditions
Source: Front Microbiol. 2016 Dec 5;7:1959. doi: 10.3389/fmicb.2016.01959 (PMC5136563; doi:10.3389/fmicb.2016.01959)
Supplement: Supplementary file 1 [file Table1.DOCX]

**Table S1.** Kinetic parameters of the different fermentations performed by duplicate. Maximum fermentation rate (R), time to reach the 10, 50 and 75% of the final density (referred as t10, t50 and t75, respectively) and residual sugar and acetic acid concentrations in g/L.

| **Samples** | **R** | **t10** | **t50** | **t75** | **Acetic ac.** | **Res. Sugar** |
| --- | --- | --- | --- | --- | --- | --- |
| 300N200S-C | 27.25 | 0.58 | 2.33 | 3.41 | 1.35 | 0.0 |
| 300N200S-C | 28.05 | 1.58 | 2.33 | 3.42 | 1.35 | 0.0 |
| 300N200S-0D | 21.70 | 0.74 | 2.58 | 3.72 | 1.02 | 4.9 |
| 300N200S-0D | 20.75 | 1.74 | 2.55 | 3.71 | 1.02 | 4.6 |
| 300N200S-1D | 12.85 | 0.86 | 3.45 | 5.07 | 1.10 | 0.0 |
| 300N200S-1D | 13.20 | 1.86 | 3.47 | 5.11 | 1.10 | 0.0 |
| 300N200S-2D | 10.98 | 1.08 | 4.04 | 5.89 | 0.63 | 0.0 |
| 300N200S-2D | 11.03 | 2.08 | 4.05 | 5.92 | 0.63 | 0.0 |
| 300N200S-5D | 17.47 | 0.65 | 2.82 | 4.17 | 0.96 | 10.2 |
| 300N200S-5D | 17.57 | 1.65 | 2.81 | 4.16 | 0.96 | 10.7 |
| 300N240S-C | 17.45 | 0.27 | 2.24 | 3.47 | 0.72 | 0.2 |
| 300N240S-C | 17.45 | 1.27 | 2.24 | 3.47 | 0.72 | 0.2 |
| 300N240S-0D | 15.78 | 0.88 | 3.44 | 5.04 | 1.70 | 5.5 |
| 300N240S-0D | 15.88 | 1.88 | 3.43 | 5.02 | 1.70 | 5.0 |
| 300N240S-1D | 10.33 | 0.13 | 3.75 | 6.01 | 0.94 | 2.6 |
| 300N240S-1D | 10.25 | 1.13 | 3.72 | 5.98 | 0.94 | 2.8 |
| 300N240S-2D | 8.87 | 0.67 | 5.80 | 9.00 | 1.04 | 0.0 |
| 300N240S-2D | 8.87 | 1.67 | 5.75 | 8.96 | 1.04 | 0.0 |
| 300N240S-5D | 8.92 | 0.99 | 5.54 | 8.38 | 1.24 | 29.9 |
| 300N240S-5D | 8.65 | 1.99 | 5.50 | 8.37 | 1.24 | 30.9 |
| 100N200S-C | 12.90 | 0.49 | 3.55 | 5.46 | 0.20 | 0.0 |
| 100N200S-C | 12.75 | 1.49 | 3.29 | 5.02 | 0.20 | 0.0 |
| 100N200S-0D | 12.65 | 0.41 | 3.46 | 5.37 | 0.16 | 0.3 |
| 100N200S-0D | 12.43 | 1.41 | 3.45 | 5.36 | 0.16 | 0.3 |
| 100N200S-1D | 7.54 | 0.47 | 4.77 | 12.02 | 1.10 | 38.6 |
| 100N200S-1D | 7.50 | 1.47 | 4.72 | 11.37 | 1.10 | 43.8 |
| 100N200S-2D | 6.80 | 0.18 | 4.95 | 11.05 | 1.30 | 60.0 |
| 100N200S-2D | 6.84 | 1.18 | 4.89 | 10.85 | 1.30 | 53.8 |
| 100N200S-5D | 6.88 | 0.24 | 4.95 | 11.05 | 1.30 | 61.2 |
| 100N200S-5D | 6.70 | 1.24 | 4.91 | 10.85 | 1.30 | 57.5 |
| 100N240S-C | 15.30 | 0.95 | 3.64 | 5.31 | 0.15 | 18.0 |
| 100N240S-C | 15.13 | 1.95 | 3.62 | 5.28 | 0.15 | 19.3 |
| 100N240S-0D | 9.13 | 0.83 | 5.16 | 7.84 | 0.40 | 13.2 |
| 100N240S-0D | 9.02 | 1.83 | 5.12 | 7.83 | 0.40 | 14.0 |
| 100N240S-1D | 8.22 | 0.95 | 5.76 | 15.02 | 1.50 | 50.4 |
| 100N240S-1D | 8.30 | 1.95 | 5.63 | 14.03 | 1.50 | 51.1 |
| 100N240S-2D | 4.65 | 1.08 | 9.58 | 16.51 | 1.40 | 45.0 |
| 100N240S-2D | 4.67 | 2.08 | 9.35 | 14.06 | 1.40 | 40.4 |
| 100N240S-5D | 5.22 | 8.65 | 8.65 | 15.32 | 1.60 | 68.3 |
| 100N240S-5D | 5.32 | 9.65 | 8.45 | 13.16 | 1.60 | 64.4 |
