# Supplementary material for: Saccharomyces and non-Saccharomyces Competition during Microvinification under Different Sugar and Nitrogen Conditions
Source: Front Microbiol. 2016 Dec 5;7:1959. doi: 10.3389/fmicb.2016.01959 (PMC5136563; doi:10.3389/fmicb.2016.01959)
Supplement: Supplementary file 2 [file Table2.DOCX]

**Table S2.** Pearson correlation values between the residual sugar and the rest of fermentation parameters. The values in bold are different from 0 with a significance level alpha = 0.05.

| Variables | Residual sugar |
| --- | --- |
| R | **-0.654** |
| t10 | 0.441 |
| t50 | **0.691** |
| t75 | **0.863** |
| Acetic acid | **0.518** |
| N | **-0.639** |
| S | 0.124 |
