# Supplementary material for: Saccharomyces and non-Saccharomyces Competition during Microvinification under Different Sugar and Nitrogen Conditions
Source: Front Microbiol. 2016 Dec 5;7:1959. doi: 10.3389/fmicb.2016.01959 (PMC5136563; doi:10.3389/fmicb.2016.01959)

Figure S1: Fermentation kinetics of the different inoculation strategies performed under four different nutrient conditions, (A) 300N-200S, (B) 300N-240S, (C) 100N-200S and (D) 100N-240S. The solid line shows the evolution of the fermentation measured by density (g/L) and the dotted line assessed by plate culturing in YPD (CFU/mL). The line color corresponds to each fermentation strategy: blue, co-inoculated fermentation; red, inoculation of *S. cerevisiae* at 24 hours; green, inoculation of *S. cerevisiae* at 48 hours; orange, inoculation of *S. cerevisiae* at 5 days, and black; control fermentation with only *S. cerevisiae*. Standard deviations were always lower than 10% and have been avoided in the figure for clarity.

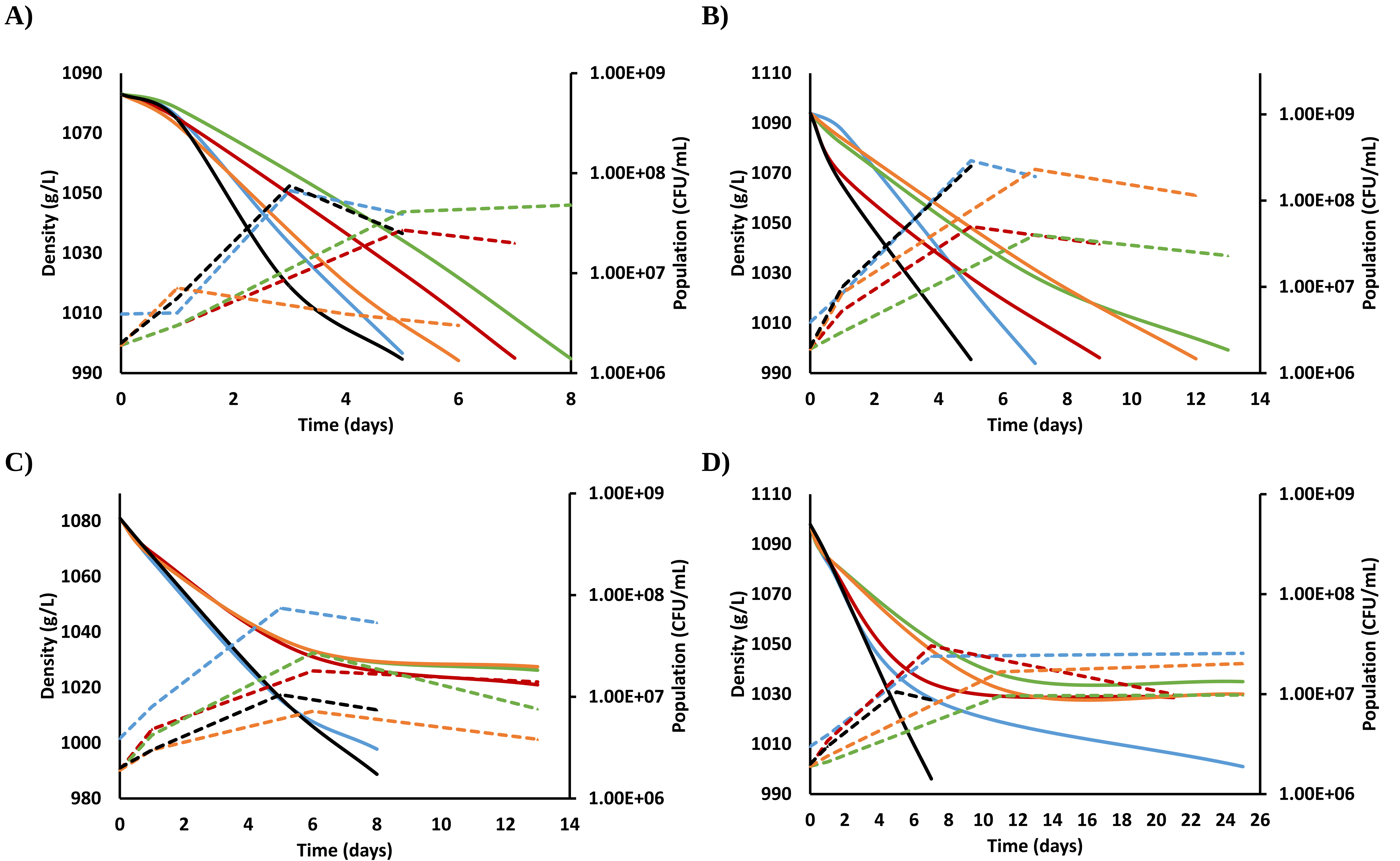

Supplement: Supplementary file 3 [file Image1.pdf]
